# Visual Learning Alters the Spontaneous Activity of the Resting Human Brain: An fNIRS Study

**DOI:** 10.1155/2014/631425

**Published:** 2014-08-28

**Authors:** Haijing Niu, Hao Li, Li Sun, Yongming Su, Jing Huang, Yan Song

**Affiliations:** ^1^State Key Laboratory of Cognitive Neuroscience and Learning and IDG/McGovern Institute for Brain Research, Beijing Normal University, Beijing 100875, China; ^2^Center for Collaboration and Innovation in Brain and Learning Sciences, Beijing Normal University, Beijing 100875, China; ^3^Peking University Sixth Hospital/Institute of Mental Health, Key Laboratory of Ministry of Health, Peking University, Beijing 100191, China

## Abstract

Resting-state functional connectivity (RSFC) has been widely used to investigate spontaneous brain activity that exhibits correlated fluctuations. RSFC has been found to be changed along the developmental course and after learning. Here, we investigated whether and how visual learning modified the resting oxygenated hemoglobin (HbO) functional brain connectivity by using functional near-infrared spectroscopy (fNIRS). We demonstrate that after five days of training on an orientation discrimination task constrained to the right visual field, resting HbO functional connectivity and directed mutual interaction between high-level visual cortex and frontal/central areas involved in the top-down control were significantly modified. Moreover, these changes, which correlated with the degree of perceptual learning, were not limited to the trained left visual cortex. We conclude that the resting oxygenated hemoglobin functional connectivity could be used as a predictor of visual learning, supporting the involvement of high-level visual cortex and the involvement of frontal/central cortex during visual perceptual learning.

## 1. Introduction

Perceptual learning (PL) refers to the relatively permanent modification of perception and behavior following a sensory experience. The orientation discrimination task is one of the most intensively studied PL tasks. In this task, subjects need to decide whether a grating or a line is tilted clockwise or anticlockwise with respect to the reference. Performance on this task dramatically improves with practice; moreover, this learning effect is specific to the position and orientation of the stimuli [1]. In typical PL models, this specificity is interpreted as an indicator of the retinotopic early visual cortical locus of learning, where different orientations are processed separately [2–4]. Indeed, significant modulation of learning on V1/V2 activity has been found in single-unit recording studies of animals and functional magnetic resonance imaging (fMRI) studies in humans [5, 6]. On the other hand, some researchers have proposed that visual PL occurs at the middle visual stages, such as the extrastriate cortex including V2–V4, where neurons are characterized by both orientation/location selectivity and more complex properties [7, 8]. Recent neuroimaging studies even proposed that the higher central mechanism, rather than the early visual processing itself, may account for orientation discrimination learning [9–11]. Therefore, the emerging view is that a single cortical area or process is not likely to be exclusively responsible for PL. Perceptual learning might be a refinement of synergistic processes in multiple stages and cortical areas, including those dedicated to sensory processing, engaged in top-down control, and involved in decision-making and memory. This hypothesis was consistent with two recent works, which showed that the resting-state blood oxygenation level-dependent (BOLD) signal functional connectivity and directed mutual interaction between trained visual cortex and frontal-parietal areas involved in the control of spatial attention were significantly modified after extensive practice on a shape-identification task [11, 12]. Critically, postlearning modulations in functional connectivity correlated with individual measures of improvement. These findings suggest that the change in spontaneous functional connectivity could encode or support the encoding of behavioral visual learning.

In the present study, we investigated whether and how classical orientation discrimination learning modified the resting oxygenated hemoglobin (HbO) functional connectivity brain activity by using functional near-infrared spectroscopy (fNIRS). We acquired resting-state fNIRS before and after intense training on the classical orientation discrimination task at a specific location in the right visual field. Compared with the fMRI that records signals based on the paramagnetic properties of deoxygenated hemoglobin (HbR), fNIRS detects changes in the optical properties of the cortical surface mediated by variations in local hemodynamic activity and can estimate variations in the concentration of both HbR and HbO at a higher temporal resolution. As an emerging neuroimaging tool, fNIRS has been successfully used to identify functional connectivity during resting-state brain activity [13–19]. If functional connectivity is related to the neural changes that occur during the orientation discrimination learning, then training-specific plasticity in functional connectivity should be observed.

## 2. Materials and Methods

### 2.1. Participants

Thirty undergraduate and graduate students (22 males; 20–25 years old) with normal or correct-to-normal vision participated in this study as paid volunteers. Subjects were randomly arranged into two groups: the “training” group (six males and nine females) and the “control” group (eight males and seven females). In addition to these thirty subjects, two subjects were excluded because of the low signal-to-noise ratio in several channels (less than 25% of the mean signal-to-noise ratio of all the channels) due to failures in source/detector placement. Four subjects were excluded due to large head movement during their fNIRS recording [15, 16]. All of the subjects signed the informed consent before the experiment, and the experimental protocol was approved by the Beijing Normal University Institutional Review Board.

### 2.2. Stimuli and Procedure

**Figure 1:**
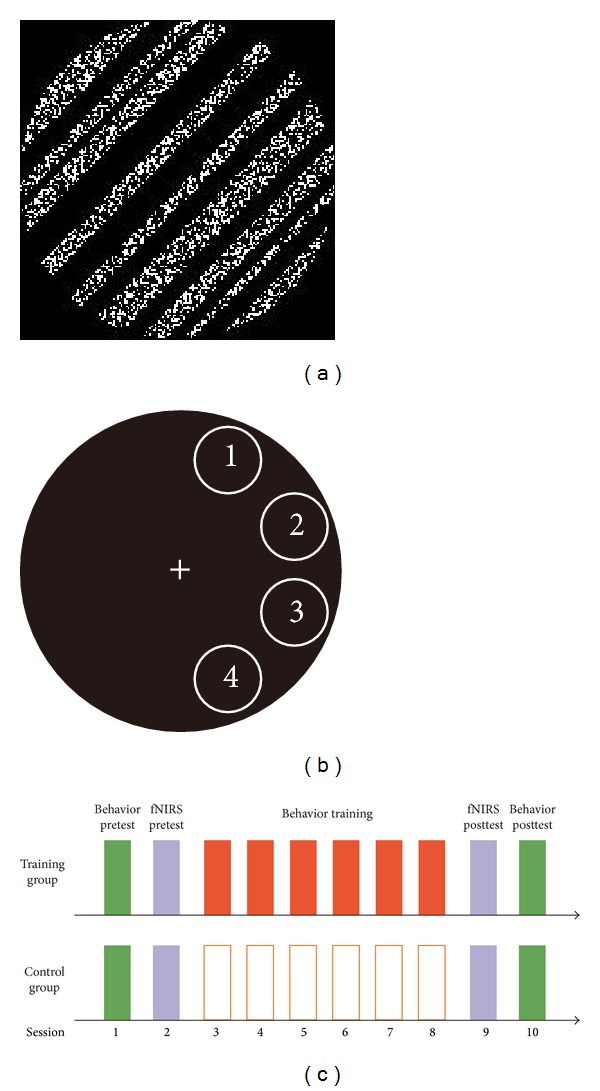
(a) Example of the stimulus used in the current study. (b) The stimulus presented at one of the four locations in the right visual field and the location of the grating were fixed for each participant during the whole experiment. The four locations were counterbalanced across participants. (c) Experiment design for the “training” group and “control” group.

The stimuli were generated using MATLAB with the Psychtoolbox extension and were presented on a 21-inch ViewSonic G220f color monitor (1024 pixel × 768 pixel resolution, 0.39 mm × 0.39 mm pixel size, 120 Hz frame rate, and 50 cd/m^2^ mean luminance). The stimulus, called a “solid noisy grating,” was a circular field (diameter = 3.8°) consisting of one-dimensional white noise (white and black bars with width varying from 0.077° to 0.312° which were reset in each trial; Figure 1(a)). Each trial started with the presentation of a white fixation cross at the center of the screen for 500 ms. Subsequently, a “solid noisy grating” would appear in one of the four locations centered at 5° eccentricity from the fixation in the right visual field for 100 ms. The location of the grating was fixed for each participant during the whole experiment, and the four locations were counterbalanced across participants. Finally, an infinite central cross was presented until a response was detected. Subjects were instructed to judge whether the grating orientation had a more clockwise or anticlockwise orientation relative to 36° by pressing one of the two buttons with their right hand. Auditory feedback would be given if the response was incorrect. A standard 3-down-1-up staircase was used in the grating orientation discrimination threshold testing, which resulted in a 79.4% convergence level. That is, when subjects' responses were correct for 3 consecutive trials, the difference between the grating and the reference 36° was decreased by one step. When subjects provided an incorrect response, the difference between the grating and the reference 36° was increased by one step. The steps of the staircase were separated by 0.05 log units. If the orientation variation trend changed, then there was a reversal. Each staircase consisted of ten reversals, four preliminary reversals and six experimental reversals. The geometric mean of the experimental reversals was taken as the threshold for each staircase, and the average of all of the thresholds in one session was taken as the threshold of this session. Therefore, the threshold in our study means the lowest difference angle at which the grating orientation can be discriminated from the reference 36°. To make sure that subjects focused their eyes on the fixation throughout the experiment, an EyeLink 1000 Long Range Mount system was used to monitor their eye movement during pretest, training, and posttest. In addition, a piece of black cardboard with a circular aperture (diameter = 17°) was used to cover the monitor to exclude the possibility that subject could utilize the edges of the monitor to perform the orientation discrimination task. Experiments were performed in a dimly lit room. Subjects sat in a chair with their heads on a chin-and-head rest to keep still at a distance of 1 m from the monitor.

For the “training” group, the experiment consisted of nine sessions on nine successive days (Figure 1(c)). First, subjects performed a behavioral pretest in session 1 (including five staircases of an orientation discrimination task) and a resting fNIRS pretest in session 2 (12 min). Then, subjects were trained with the same orientation discrimination task in session 3 to session 7 (including 100 staircases in total, 20 staircases for each session). After five sessions of training, subjects had a resting fNIRS posttest in session 8 (the same as the fNIRS pretest) and a behavioral posttest in session 9 (the same as the behavioral pretest in session 1). All training and test sessions were performed in a 36° orientation.

To determine whether the changes in functional connectivity were induced by the training or the simple passage of time, participants in the “control” group did not take part in any type of training and participated in only the same behavioral and fNIRS measurements in the pre- and posttests (session 1, session 2, session 8, and session 9; Figure 1(c)).

### 2.3. fNIRS Measurement

**Figure 2:**
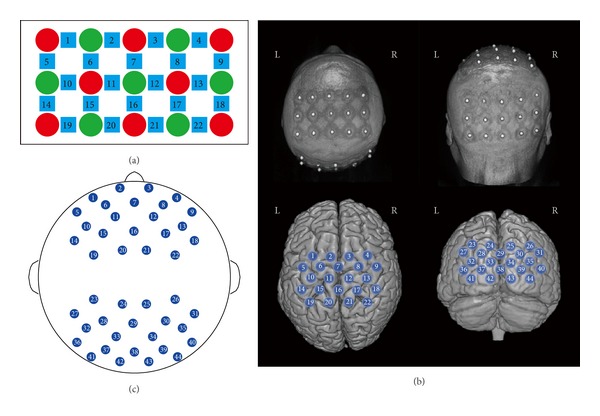
(a) The 3 × 5 optode probe set with seven detectors (green) and eight sources (red), resulting in 22 channels (blue). (b) Cerebral projections of light sources and detectors overlaid on the participants' scalp (top). The two 22 channels (total 44 channels) were fitted on the frontal/central and posterior cortex of the head, respectively (bottom). (c) The total 44 channels on the head model.

The resting-state fNIRS measurements were conducted using a continuous wave system (ETG-4000, Hitachi Medical Co., Japan). The system generated two wavelengths of near-infrared light (690 and 830 nm) and collected the hemoglobin concentration at 10 Hz sampling rate. Two 3 × 5 optode probe sets (consisting of 7 photodetectors and 8 light emitters, 3 × 3 cm probe configuration) were used to produce 44 measurement channels to allow for the measurement of frontal/central and posterior cortices (Figure 2). Specifically, channel 16 was placed at Cz according to the international 10–20 system, and the middle point of channels 42 and 43 was placed at the inion. The probe sets were examined and adjusted to ensure the consistency of the positions across the participants.

All fNIRS channels were marked on one participant's scalp by vitamin E capsules which are visible in structural MR imaging (Figure 2(b), top). NIRS-SPM was used to project the measurement channels onto the cortical surface and to further determine the anatomical localization of each fNIRS measurement channel (Figure 2(b), bottom).

During the fNIRS measurements, subjects sat in a comfortable chair with their eyes fixed on a black cross presented on a gray background for 12 min. They were instructed to relax and keep motionless as much as possible.

The structural MRI data were acquired using a SIEMENS TRIO 3-Tesla scanner in the Imaging Center for Brain Research, Beijing Normal University. During MRI data acquisition, the participant was supine in the MRI scanner with the two probes placed on the subject's head, which was fixed by straps. The T1-weighted structural image was acquired using a magnetization-prepared rapid gradient echo (MPRAGE) sequence: 206 slices, TR = 2530 ms, TE = 3.39 ms, slice thickness = 1 mm, FA = 7°, FOV = 256 × 256 mm^2^, and in-plane resolution = 256 × 256.

### 2.4. Data Processing

The psychophysical data of two groups were analyzed with a repeated-measure ANOVA with the two factors being “group” (training versus control group, between-subject factor) and “test” (pretest versus posttest, within-subject factor).

For fNIRS data processing, optical data were first converted from relative changes in light intensities to HbO and HbR for each channel based on the modified Beer-Lambert law using HomER [20]. The converted data were visually checked and excluded from subsequent processing once the pre- or posttest data included large motion artifacts or a low signal-to-noise ratio. Then, a band-pass filter (0.009–0.08 Hz) was adopted to remove the high-frequency physiological noises and low-frequency baseline drifts. Finally, for each subject, we cut 10 min low-frequency oxyhemoglobin signals to be used as functional connectivity analysis at resting state.

For each subject and each test, we calculated the Pearson correlation coefficients of every two channels' time course to produce a correlation matrix. Then, the coefficients were *z*-transformed through the function  *z* = (1/2)log⁡⁡((1 + *r*)/(1 − *r*)). Next, we applied a one-tailed paired* t*-test between the pretest and posttest for each group to determine whether there were significant changes in functional connectivity. Among them, we further identified the ones which could significantly predict the behavioral improvement by computing correlation coefficient.

## 3. Results

### 3.1. Psychophysical Data

**Figure 3:**
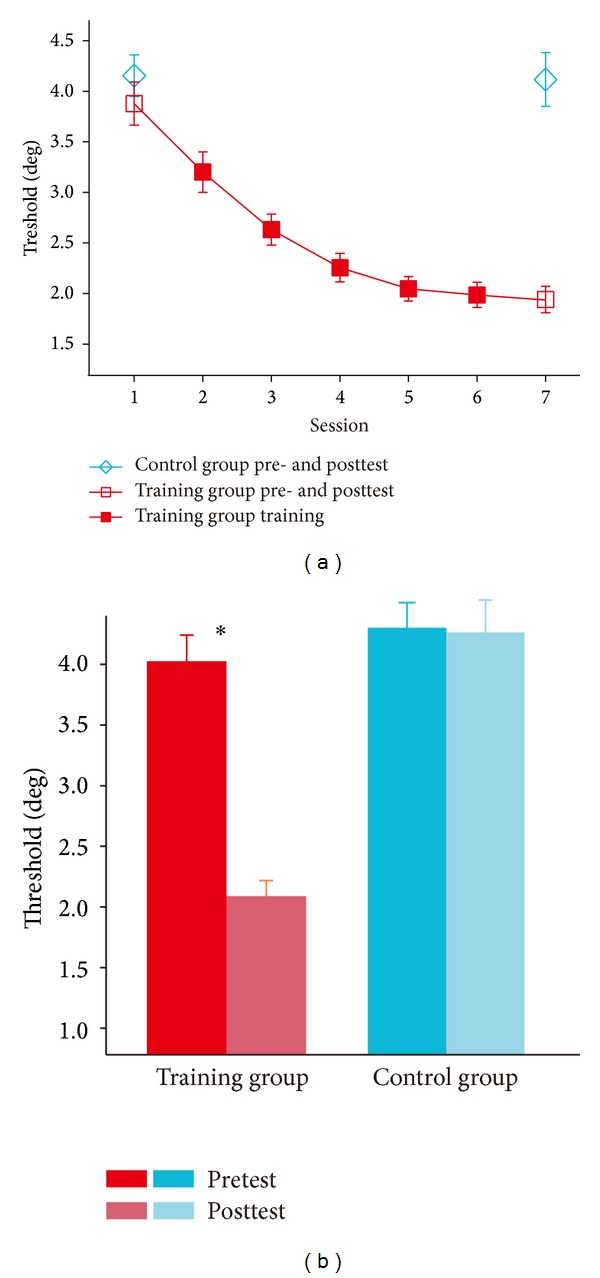
(a) The learning curves of the “training” group (solid red squares), the pre- and posttest thresholds for the “training” group (hollow red squares), and the “control” group (hollow blue diamonds). (b) The pre- and posttest thresholds for the “training” group (red columns) and the “control” group (blue columns).

Figure 3 shows the learning curve and mean data obtained from the psychophysical threshold measured in the training and control groups. The 2 × 2 repeated-measure ANOVA shows that the main effect of the group was significant (F(1, 59) = 7.958, *P* < 0.05), and the interaction of group × test was also significant (F(1, 59) = 4.772, *P* < 0.05). Simple effect analysis showed that, for the “training” group, psychophysical thresholds in the behavioral posttest were significantly lower than those in the behavioral pretest (*P* < 0.01, Figure 3(b)). For the “control” group, however, psychophysical thresholds did not significantly differ between two tests (*P* > 0.9; Figure 3(b)). In addition, the “training” and “control” groups had similar pretest thresholds (*P* > 0.5), Therefore the observers in two groups were homogeneous in the behavioral dimension before the training.

### 3.2. RSFC Changes

**Figure 4:**
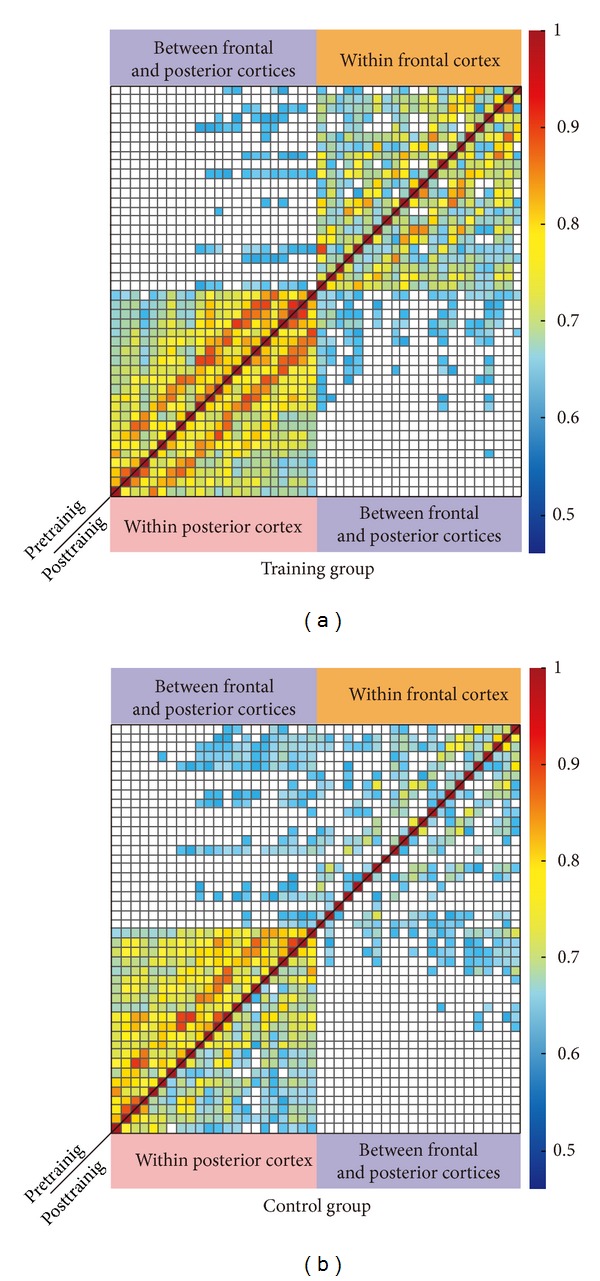
Pre- and posttest spontaneous HbO functional connectivity correlation matrix for the “training” group (a) and the “control” group (b). Color bar indicates correlation values for each channel. Note the stability of the correlation matrix across tests, indicating that within-probe functional connectivity is very stable over time.

For the HbO responses, the functional connectivity correlation matrix of the pre- and posttest is illustrated in Figure 4. Regardless of the group, it was obvious that both pre- and posttests had stronger functional connectivity within probes than between the two probes.

**Figure 5:**
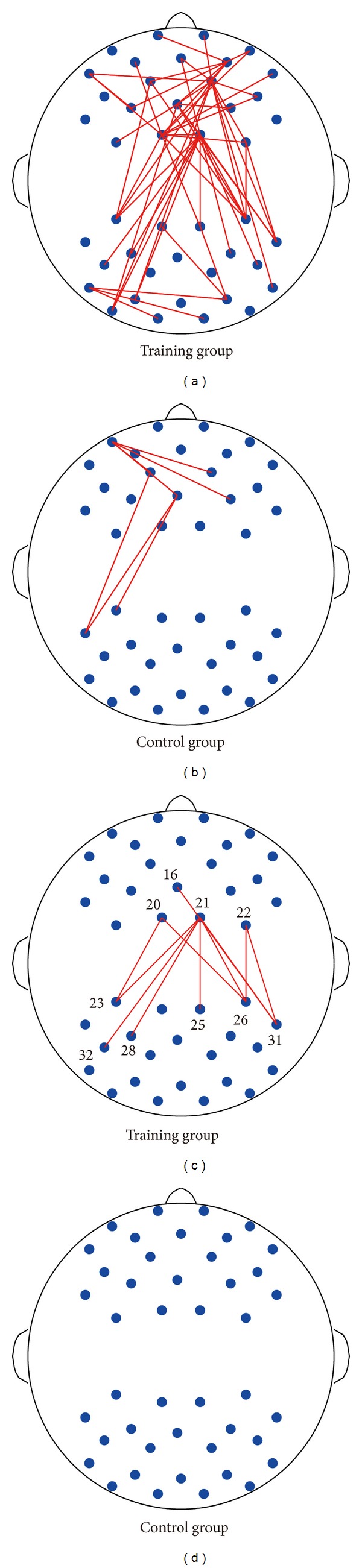
Clusters for the significantly strengthened functional connectivity after training (a, b). Clusters for which the strengthened functional connectivity significantly predicted behavioral improvement (c, d).

Then, we examined the changes of the connectivity patterns induced by perceptual learning. Although the functional connectivity patterns for the “training” group showed a high degree of similarity between the pre- and posttests, the one-tailed* t*-test results showed that the strength of a large number of connections was improved after learning (Figure 5(a)), and most were between-probes rather than within-probes. It is also worth noting that the changes in functional connectivity were centered at the right superior frontal area (CH12) and the right postcentral area (CH21). For the “control” group, however, the change pattern of functional connectivity was less obvious and the strength of only a few connections was significantly improved (Figure 4(b)).

We further checked whether the connectivity strength was decreased; however, no such connectivity was found in both groups.

### 3.3. Correlation between RSFC Changes and Learning

**Figure 6:**
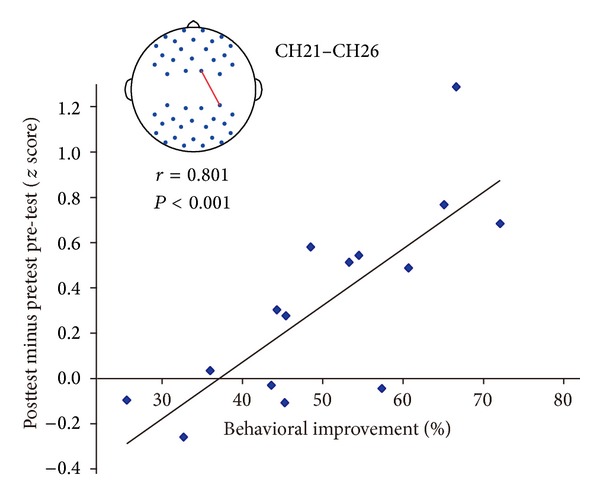
The strengthened CH21–CH26 functional connectivity significantly predicted behavioral improvement.

For each functional connectivity that changed significantly after learning, correlation analyses were carried out to determine if there was a relationship between the change in behavioral performance (the threshold difference between post- and pretest) and the change in functional connectivity (the strength difference between post- and pretest). For the “training” group, the changes in functional connectivity between the right postcentral area (CH21) and the following regions significantly predicted participants' behavioral performance: left middle occipital gyrus (CH32), right middle occipital gyrus (CH26), left superior occipital gyrus (CH23, CH28), right superior occipital gyrus (CH25), and right angular gyrus (Figures 5(c) and 6; Tables 1 and 2). In addition, the strength of functional connectivity between the right postcentral gyrus (CH22) and right middle occipital gyrus (CH26), between the right postcentral gyrus (CH22) and the right angular gyrus (CH31), between the left precuneus (CH20) and occipital lobe (CH23, CH26), and between right paracentral lobule (CH16) and right angular (CH31) also positively correlated with the behavioral improvement. These measures involved the channels adjacent to CH21 (Figures 5(c) and 6; Tables 1 and 2). All of these results indicated that the stronger the long functional connectivity became, the more the improvement the subjects would make. More importantly, although the gratings were only presented to the right visual field (hence the visual information was mainly projected to the left visual cortex) during the whole training, the connectivity between frontal channels and right posterior channels not directly stimulated by the grating stimuli was also significantly increased. Furthermore, those changes had a strong correlation with behavioral improvement.

For the “control” group, however, none of the changes in functional connectivity were reliably correlated with the behavioral performance improvement (Figure 5(d)).

Lastly, we analyzed HbR concentration data by using the same methods applied to the HbO. Neither the significantly changed connectivity nor reliable correlation between the HbR and behavioral performance was found in either group, supporting the conclusion that HbO was the most sensitive indicator of changes in regional cerebral blood flow in fNIRS measurements [21].

## 4. Discussion

By investigating the spontaneous HbO connectivity changes in observers who were trained with grating orientation discrimination, we demonstrated that orientation discrimination learning is associated with stronger functional connectivity. Critically, these changes correlated with the individual degree of perceptual learning, suggesting that visual perceptual learning can change the pattern of spontaneous cortical activity between different brain networks in specific ways. The specific learning-related modulation in resting HbO connectivity was not induced by the simple passage of time because these effects disappeared in the control group.

Visual perceptual learning is often taken as evidence of neural plasticity in the retinotopic early visual cortex [1, 22, 23]. However, recent psychophysical studies suggest that perceptual learning is a high-level learning process beyond the retinotopic early visual areas [24–28]. Our results showed that when the task is performed only at the right visual field, the functional connectivity was significantly increased in both left and right high-level visual cortex. This nonretinotopic effect suggests the involvement of top-down influence. Single-unit and fMRI studies have shown that not only the retinotopic early visual cortex, but also the nonretinotopic higher brain areas that are more related to attention and decision-making are involved in visual discrimination [29, 30]. Recent neurophysiological and fMRI evidence has further shown that the brain areas responsible for decision-making, such as LIP and ACC, are also involved in visual perceptual learning [10, 31]. Therefore, it is not surprising to find that the increased functional connectivity mainly involved the frontal/central cortex and high-level visual cortex in this study, suggesting the top-down modulation in higher-order decision-making or attention systems [32–34].

Resting-state measures have been correlated with individual performance variability in several cognitive domains, such as working memory [35], executive control [36], reading competence [37], and face perception [38]. In the domain of visual learning, fMRI has been used to examine the mechanism of learning-induced changes in resting functional connectivity. For example, Lewis et al. showed that resting-state blood oxygenation level-dependent (BOLD) functional connectivity between visual cortex and task-relevant cortical networks changed after learning on a shape-identification task [11]. Different from our results, the BOLD functional connectivity between trained visual cortex and dorsal attention regions became weaker after learning, and this decrement in functional connectivity strength was positively correlated with behavioral improvement.

Our results provide evidence for the functional role of spontaneous HbO coherence in cortical networks as identified by fNIRS. The finding that most relevant changes in our study occur between, rather than within, networks indicates that this signal may be especially important in linking large-scale cortical networks, which is consistent with the work of Lewis et al. [11]. This is also consistent with the suggestion that coherence is related to low-frequency fluctuations of neuronal activity that are deemed very important for long-distance cortical communication [39].

In conclusion, we used fNIRS to explore RSFC changes during visual learning and proved that resting oxygenated hemoglobin functional connectivity could be used as a predictor of visual learning. Our results provide further support that the coordinated activation of cortical networks during behavior shapes the organized pattern of correlated spontaneous activity at rest. The individual degree of orientation discrimination learning was mainly related to the changes in functional connectivity between high-level visual cortex and frontal/central association areas but not within portions of visual cortex.

## Figures and Tables

**Table 1:** The MNI coordinate, region, and BA for some NIRS channels.

Channel	MNI			Region	BA
	*x*	*y*	*z*		
CH16	4	−29	78	Paracentral_Lobule_R	4
CH20	−9	−45	79	Precuneus_L	5
CH21	15	−43	80	Postcentral_R	5
CH22	36	−41	71	Postcentral_R	3
CH23	−27	−89	39	Occipital_Sup_L	19
CH25	20	−92	38	Occipital_Sup_R	19
CH26	41	−81	40	Occipital_Mid_R	19
CH28	−16	−98	29	Occipital_Sup_L	18
CH31	52	−75	32	Angular_R	39
CH32	−27	−98	21	Occipital_Mid_L	18

**Table 2:** Clusters for which the strengthened functional connectivity can predict the behavioral improvement.

Connectivity	Correlation with behavioral improvement	
	*r*	*P* value
CH21–CH26	0.801	<0.001
CH21–CH23	0.791	<0.001
CH21–CH31	0.772	<0.001
CH20–CH23	0.665	<0.01
CH21–CH28	0.637	<0.05
CH20–CH26	0.631	<0.05
CH21–CH25	0.598	<0.05
CH21–CH32	0.596	<0.05
CH22–CH26	0.591	<0.05
CH22–CH31	0.572	<0.05
CH16–CH31	0.556	<0.05
